# A Quality Improvement Project on Participant Characteristics, Engagement and Outcomes of the Mentalisation-Based Therapy Introduction (MBT-I) Programme Within Leicestershire Partnership NHS Trust

**DOI:** 10.1192/bjo.2026.11384

**Published:** 2026-06-30

**Authors:** Dafni Maria Gavotsi, Yamini Ram

**Affiliations:** 1 Leicester Royal Infirmary, Leicester, United Kingdom; 2 Leicestershire Partnership NHS Trust, Leicester, United Kingdom

## Abstract

**Aims:**

Mentalisation-Based Therapy-Introduction (MBT-I) is a brief, structured psychological intervention designed to enhance reflective functioning and emotional regulation, and to prepare individuals for longer-term psychotherapeutic work. Despite increasing use across NHS services, evidence relating specifically to MBT-I remains limited. This quality improvement project (QIP) aimed to evaluate the characteristics, engagement, and outcomes of participants enrolled in MBT-I groups within Leicestershire Partnership NHS Trust (LPT). Objectives included describing demographic and clinical characteristics, examining referral pathways and engagement, assessing post-intervention outcomes, and identifying service gaps and future needs for MBT group provision.

**Methods:**

A retrospective audit was conducted across three MBT-I cohorts delivered in October 2023, July 2024, and February 2025. Referrals were accepted from primary care, secondary mental health services, Central Access Point, and self-referral pathways. Forty-eight individuals were considered for MBT-I. Groups were delivered weekly over 10–13weeks, co-facilitated by two MBT-trained clinicians. Data were collected at pre-intervention, during treatment, and post-completion using routinely recorded clinical information. Variables included demographics, mental health history, risk and safeguarding data, attendance, completion rates, and onward referrals.

**Results:**

Participants represented a clinically complex cohort (mean age 40.2 years; 77.1% female). Historical risk to self was present in 66.7% and 91.7% had previously engaged in psychological therapies. Most participants (85.4%) had no prior psychiatric admissions. Engagement was good, with a median attendance of 8.5 sessions with attendance ranging from 0 to 13 and low rates of non-attendance. Inpatient admissions during MBT-I were rare (2.1%). Following MBT-I, 20.8% completed and were referred for further psychological therapy, 12.5% completed and required no further intervention, and 4.2% were signposted to alternative services. A minority (22.9%) did not complete the programme, while 37.5% did not commence following assessment. Referrals predominantly originated from secondary mental health services, with minimal primary care referrals.

**Conclusion:**

MBT-I appears to be a feasible, acceptable, and resource-efficient intervention for individuals with complex emotional and interpersonal difficulties. Findings demonstrate good engagement and low inpatient admission rates, supporting its safe delivery within high-risk populations. MBT-I may function both as a gateway to longer term psychological therapy and, for some individuals, as a sufficient standalone intervention. Non-commencement and drop-out highlight the importance of robust assessment and exploration of barriers to engagement. Future service development should prioritise routine outcome measurement, improved data quality, and clearer integration of MBT-I within stepped-care psychological therapy pathways. These findings align with NICE guidance and support further evaluation,expansion, and integration of MBT-I provision within NHS psychotherapy services nationally sustainably.

